# The association between chronic spontaneous urticaria and HLA class I and class II antigen

**DOI:** 10.3906/sag-1907-159

**Published:** 2020-08-26

**Authors:** Neslihan DOĞAN, Songül ÇİLDAĞ, Çiğdem YENİSEY, Taşkın ŞENTÜRK

**Affiliations:** 1 Department of Internal Medicine, Faculty of Medicine, Aydın Adnan Menderes University, Aydın Turkey; 2 Department of Immunology and Allergy, Faculty of Medicine, Aydın Adnan Menderes University, Aydın Turkey; 3 Department of Biochemistry, Faculty of Medicine, Aydın Adnan Menderes University, Aydın Turkey

**Keywords:** Chronic spontaneous urticaria, genetic, HLA antigens

## Abstract

**Background/aim:**

Chronic spontaneous urticaria (CSU) is a chronic disease with an unknown etiology. In human leukocyte antigen (HLA) system, the association of class I and class II antigens with autoimmune diseases has been identified and HLA antigens that have a tendency to or can prevent chronic urticaria have been studied. The aim of this study is to investigate the association between chronic spontaneous urticaria and HLA class I and class II antigens.

**Materials and methods:**

A total of 80 subjects, 40 patients with CSU and 40 healthy individuals were enrolled in the study. DNA sample isolation from blood was primarily done by the real-time polymerase chain reaction (RT-PCR) technique for the first time. Using HLA SSP Typing Kit (ROSE Cat. No: 800118) PCR technique, HLA-A, B, C, DRB and DQB alleles from DNA samples were analyzed.

**Results:**

The mean age was 36.80 ± 9.48 years and the duration of the disease was 4.26 ± 5.18 years. Among the HLA class I and class II antigens, HLA-A was detected significantly more often in the control group (P = 0.039). HLA-DRB1 was more often detected in the CSU group but no statistical difference (P > 0.05).

**Conclusion:**

It can be considered that HLA-DRB1 may have a tendency to CSU, while HLA-A might prevent the disease.

## 1. Introduction

Urticaria is a cutaneous vascular reaction characterized by formation erythematous, edematous, itchy papules and plaques with varying sizes involving the upper and middle dermis, with mast cells playing a role in its pathogenesis. If this vascular reaction involves deep dermis, subcutaneous tissues, or mucosae, it is called angioedema. While urticarial lesions disappear within 24 h, disappearance of angioedema can take up to 72 h. Lesions are defined as acute urticaria lasting less than 6 weeks and as chronic urticaria (CU) lasting 6 weeks or longer. There are 2 subtypes of chronic urticaria; chronic spontaneous urticaria (CSU) and inducible urticaria. Whereas an etiologic cause can be detected frequently in patients with acute urticaria, any triggers have not been found in approximately 80% of patients with chronic urticaria [1,2]. Although many studies have been conducted on the etiology and pathogenesis of CSU, its reason and mechanism still remain unknown.

In recent years, studies on the etiology of chronic urticaria have noted autoimmunity in approximately 1/3 of cases [3–6]. The association of both class I and class II antigens with autoimmune diseases has been described in the human leukocyte antigen (HLA) system, also known as major histocompatibility complex (MHC) [7,8]. There are some studies investigating the association between chronic spontaneous urticaria and HLA antigens. In these studies, it has been shown that some alleles are susceptible to disease and some alleles may be protective [9–16].

The objective of this study is to examine the relation between chronic spontaneous urticaria and HLA class I and class II antigens.

## 2. Material and methods

### 2.1. Patients and study design

The cross-sectional study was included a total of 80 subjects; 40 patients aged 18 years and above who were diagnosed as CSU patients between August 2017 and December 2017 in the Immunology–Allergy Clinic, and 40 healthy individuals as a control group with no history of urticaria. 

All participants were informed of the nature of the study and written consent was obtained from each. 

For this research, approval was received from the Ethics Committee for Noninvasive Clinical Studies at the Aydın Adnan Menderes University School of Medicine (No: E.44836). The study was conducted in accordance with the principles of the Declaration of Helsinki. 

A detailed history and physical examination were obtained from each patient, with use of medications, diet, triggering physical factors, history of autoimmune disease, neoplastic disease, and infectious conditions. In the laboratory, complete blood count, erythrocyte sedimentation rate, C-reactive protein, liver function tests, renal function tests, antinuclear antibody, serological tests for hepatitis B and C, stool parasite screening, urine analysis, complement 3 (C3), complement 4 (C4), thyroid stimulating hormone (TSH), antithyroglobulin (anti-TG), antithyroid peroxidase antibody (anti-TPO), and total IgE were measured. Patients who had a history of autoimmune disease (such as connective tissue disease, vasculitis, and thyroiditis), malignancy, infectious conditions, and triggering factor (such as diet, medication, and triggering physical factors) or detected during the evaluation of the patients were excluded from the study.

### 2.2. DNA isolation and determination of HLA antigens

HLA antigens were performed on blood samples and DNA samples isolation from blood was primary done by real-time polymerase chain reaction (RT-PCR) technique for the first time.

Isolation of DNA samples from blood was conducted with the commercial kit (General DNA Extraction Kit, Cat. No: was ab156893, Abcam). DNA concentrations in the isolated 80 blood samples were measured via Qubit fluorometers. Using HLA SSP Typing Kit (ROSE Cat. No: 800118) PCR technique, HLA-A, B, C, DRB, and DQB alleles were detected from DNA samples. The primers, which designed specific alleles and using with specific DNA polymerases, and the alleles, which involved with HLA subtype, were determined.

### 2.3. Statistical analyses

Power analysis was performed with 5% alpha level and 80% test power for sample size. Statistical analyses were carried out using the IBM SPSS 18.0 statistics program. The categorical ones of the descriptive data were expressed using numbers and percentages. The frequency of antigens (HLA-A, HLA-DQB and HLA-DRB1) from patients and controls were compared chi-square analysis with Fisher exact test. A P value of  <0.05 was considered statistically significant **.**

## 3. Results

A total of 40 CSU patients, 12 male and 28 female, were enrolled in this study. The mean age was 36.80 ± 9.48 years and the duration of the disease was 4.26 ± 5.18 years. The mean age of disease onset was 32.2 ± 10.2 years. Comorbid diseases were 5% diabetes mellitus, 10% hypertension. 

When HLA class 1 and class 2 antigens were compared, HLA-A was detected in 6 (15%) patients in the CSU group and 14 (35%) in the control group (P = 0.039). HLA-DRB1 was detected in 13 (32.5%) patients in the CSU group and 7 (17.5%) in the control group but there was no significant difference (P > 0.05). The frequency of HLA class 1 and 2 antigens is shown in Table 1. HLA-A*03 of the HLA-A alleles was detected to be higher in the control group (Table 2). HLA-DQB was detected in the same ratio in both groups, and suballels were detected similar ratio (Table 3). When the phenotypes of HLA-DRB1 alleles were compared, HLA-DRB1*01 was detected in a higher ratio in CSU group (Table 4).

**Table 1:** Frequency of HLA class 1 and class 2 antigens in study groups.

	CSU n= 40	Control n = 40
HLA-A**	6 (15%)	14 (35%)
HLA-B	3 (7.5%)	3 (7.5%)
HLA-C	1 (2.5%)	1 (2.5%)
HLA-DQB	9 (22.5%)	9 (22.5%)
HLA-DRB1	13 (32.5%)	7 (17.5%)
HLA-DRB2	0	0
HLA-DRB3	2 (5%)	2 (5%)
HLA-DRB4	1 (2.5%)	2 (5%)
HLA-DRB5	2 (5%)	2 (5%)

CSU: Chronic spontaneous urticaria; HLA: Human leukocyte antigen. **P< 0.05

**Table 2:** Phenotype frequency of HLA-A alleles.

	CSU n = 40	Control n = 40
HLA-A**	6 (15%)	14 (35%)
A*01	2 (5%)	2 (5%)
A*02	0	0
A*03	1 (2.5%)	6 (15%)
A*11	0	0
A*12	0	0
A*24	1 (2.5%)	1 (2.5%)
A*29	0	0
A*30	0	2 (2,5%)
A*33	1 (2.5%)	1 (2.5%)
A*74	0	0

CSU: Chronic spontaneous urticaria; HLA: Human leukocyte antigen. P< 0.05

**Table 3:** Phenotype frequency of HLA-DQB alleles.

	CSUn = 40	Controln = 40
HLA-DQB	9 (22.5%)	9 (22.5%)
DQB1 * 02	1 (2.5%)	2 (5%)
DQB1 * 0302	3 (7.5%)	2 (5%)
DQB1 * 05	1 (2.5%)	0
DQB1 * 0609	0	2 (5%)

CSU: Chronic spontaneous urticaria; HLA: Human leukocyte antigen.

**Table 4:** Phenotype frequency of HLA-DRB1 alleles.

	CSU n = 40	Control n = 40
HLA-DRB1	13 (32.5%)	7 (17.5%)
DRB1 * 01	5 (12.5%)	2 (5%)
DRB1 * 04	0	0
DRB1 * 07	2 (5%)	1 (2.5%)
DRB1 * 0901	1 (2.5%)	1 (2.5%)
DRB1 * 10	0	0
DRB1 * 11	0	0
DRB1 * 12	1 (2.5%)	0
DRB1 * 1302	2 (5%)	1 (2.5%)
DRB1 * 14	0	1 (2.5%)
DRB1* 15	0	0

CSU: Chronic spontaneous urticaria; HLA: Human leukocyte antigen.

## 4. Discussion

Chronic spontaneous urticaria is a disease of unknown etiology, usually occurring in adulthood, which is twice as common in females [4–6]. The disease usually begins in the third to fifth decades of life and the duration of the disease varies from 2 to 5 years [17–21]. In our study, in accordance with the literature; 70% of the CSU patients were female, the mean age of onset of disease was 32.2 ± 10.2 years, and the duration of disease was 4.26 ± 5.18 years. 

Currently, etiopathogenesis of chronic urticaria cannot be clearly understood and approximately 80% of patients have no etiological cause [1,2]. In studies on the etiology, autoimmunity is deemed responsible for approximately 1/3 of patients [1–6]. Although the exact mechanism of etiopathogenesis is not known, some studies support the hypothesis that HLA alleles may play a role in the pathogenesis of chronic urticaria during the initiation of immune responses [10]. It has been shown that some HLA alleles may be responsible for chronic urticaria and some alleles may be protective [22]. However, there is still no association demonstrated between specific HLA alleles and identified chronic urticaria subtypes [13]. 

There are some studies showing that HLA-A is a protective allele for chronic urticaria. Aydogan et al. [12] showed in a study conducted in Turkey that HLA-A*24 allele was higher in the control group. In other study that was done in Poland, Bozek et al. [14] reported that HLA-A*33 allele was higher in the control group. Similar to these 2 studies, it was found in our study that HLA-A was significantly higher in the control group (P = 0.039). However, in our study, HLA-A*24 and A* 33, which are among the sub alleles of HLA-A, were found to be in similar ratios in the study groups, whereas HLA-A*03 was higher in the control group. 

HLA-B, one of the HLA class I antigens, was found to be in higher ratios in patients with chronic urticaria. Similar to Bozek et al.’s [14] study that was conducted in Poland, Çoban et al. [13] also showed that HLA-B*44 allele was found to be in higher ratios in patients with CU, in Turkey. Moreover, Calamita et al. [16] in Brazil found that HLA-B*50 allele was higher in patients with CSU. In another study conducted by Calamita et al. [23], the prevalence of HLA-B*14 was found to be higher in patients with CSU with positive anti-TPO antibodies. Another study conducted in Turkey by Aydogan et al. [12] showed that the HLA-Bw4 allele is significantly higher in the chronic ordinary urticaria patient group. In our study, since the frequency of HLA-B was detected in 3 patients in each study group, the subgroup allele study was not performed. 

In studies investigating HLA class II antigens, especially HLA-DRB1*04 allele was more often detected in the patient group. In the studies conducted by Bozek et al. [14] and Öztaş et al. [10] HLA-DRB1*04; and in the study by O’Donnell et al. [9] both HLA-DRB1*04 and HLA-DQB1*0302 (DQ8) were more common in the patient group. Chen et al. [11] found that HLA-DRB1*12 and HLA-DRB1*0901 alleles were higher in CU patients. Çoban et al. [13] found that HLA-DRB1*01 and HLA-DRB1*15 alleles were predominant in CU group. Although the HLA-DRB1*15 allele was detected as a predominant allele in the chronic idiopathic urticaria (CIU) patient group in this study, it was found significantly less in the study of O’Donnell et al. [9]. In our study, as in many studies, DRB1 allele was found to be higher in the CSU group, whereas HLA-DRB1*01 was found to be in higher ratio. HLA-DRB1*04 was not detected in the patient or control groups.

An HLA class II antigen, HLA-DQ1 were found to be significantly higher in the patient group in the study performed by Aydogan et al.[12].

HLA-DQB1*06 (DQ6) allele was found to be significantly less in the CIU group in the study of O’Donnell et al. [9] and HLA-DQB1*05 allele was found to be significantly less in the CU group in the study by Chen et al. [11]. In our study, HLA-DQB was found to be in the same number in both groups and suballeles were similar.

When all these studies were evaluated collectively, different results were found in different ethnicities[7,9,10–16,22,23]. The obtained data were shown in Table 5.

**Table 5:** Frequency of HLA class I and class II Antigens in chronic urticaria.

Author	Country	Method	n-Diagnosis	HLAclass	Findings-increased alleles	Findings-decreased alleles
O’Donnell et al., 1999	U.K.	PCR-SSP	100-CIU	II	DRB1*04 DQB1*0302	DRB1*15 DQB1*06
Oztas et al., 2004	Turkey	PCR-SSP	42-CU	II	DRB1*04	No significant difference
Chen et al., 2005	China	PCR-SSP	42-CU	II	DRB1*12 DRB1*0901	No significant difference
Aydogan et al., 2006	Turkey	LCT	55-COU	I,II	BW4 DQ1	A24
Çoban et al., 2008	Turkey	PCR-SSP	40-CU	I,II	B44 DRB1*01 DRB1*15	No significant difference
Bozek et al., 2010	Poland	LCT-PCR-SSP	115-CSU	I,II	B44 DRB1*04	A33
Calamita et al., 2013	Brazil	PCR-SSO-flow cytometry	42-CSU	I,II	No differences	No significant difference

LCT: Lymphocytotoxicity assay; PCR‐SSP: Polymerase chain reaction sequence single specific primer; PCR-SSO: Polymerase chain reaction sequence specific oligonucleotide; CIU: Chronic idiopathic urticaria; CU: Chronic urticaria; CSU: Chronic spontaneous urticaria; COU: Chronic ordinary urticaria.

In the study of Çoban et al.[13], the last published study from Turkey about HLA class I and class II genotyping in patients with chronic urticaria, the autologous serum skin test was performed but was not represent any difference regarding HLA class I and II alleles. In this study, HLA-B44, HLA-DRB1*01, HLA-DRB*15 frequency was significantly higher in the patient group. There was no significant difference in HLA-A allelic distribution between the patient and control groups. However, it is not specified whether chronic urticaria patients included in the study are chronic spontaneous urticaria or chronic inducible urticaria. In addition, autoimmune diseases, malignancy, infectious conditions accompanying chronic urticaria, and triggering factor were not specified. In our study, only chronic spontaneous urticaria patients were included; patients with inducible urticaria were excluded. Patients who had a history of autoimmune disease, malignancy, infectious conditions, and triggering factors were excluded from the study.

Similar to other studies, HLA-A was higher in the control group, whereas HLA-DRB1 was higher in the CSU group in our study. According to all these results, although different results were found in terms of suballeles, HLA-DRB1 may have a tendency to CSU, while HLA-A might prevent the disease.

Epidemiological studies involving a larger number of patients may demonstrate the effect of the HLA-DRB1 allele and suballeles on the disease.

## Disclaimer/Conflict of interest

This study was founded by Aydın Adnan Menderes University Department of Scientific Research Projects (TPF-17061).

## Informed consent

All participants were informed of the nature of the study, and written consent was obtained from each.
